# Investigation of the rhythmic recruitment of tear neutrophils to the ocular surface and their phenotypes

**DOI:** 10.1038/s41598-024-57311-8

**Published:** 2024-03-25

**Authors:** Yutong Jin, Ceili Minten, Mara Jenkins, Lyndon Jones, Maud Gorbet

**Affiliations:** 1 School of Optometry and Vision Science, University of Waterloo, Waterloo, Canada; 2 Department of Systems Design Engineering, University of Waterloo, Waterloo, Canada; 3 Centre for Ocular Research and Education, University of Waterloo, Waterloo, Canada

**Keywords:** Neutrophils, Closed-eye environment, Circadian, Degranulation, NETs, Inflammation, Innate immune cells, Circadian rhythms

## Abstract

Hundreds of thousands of polymorphonuclear neutrophils (PMNs) are collected from the ocular surface upon waking, while few are harvested during daytime. This study aimed to investigate potential factors contributing to the circadian infiltration of tear PMNs, including changes in IL-8 and C5a in tears, and their phenotypes across different time points in a 24-h cycle. Tear PMNs were collected using a gentle eyewash after 2-h and 7-h of sleep (eye closure, EC) at night, after 2-h EC during the day, and towards the end of the afternoon. Significantly fewer cells were collected after 2-h EC during the day compared to 2-h EC at night. A positive correlation between IL-8 and PMN numbers existed, but not with C5a. Tear PMNs collected after 2-h EC at night were less degranulated and possessed a larger activation potential compared to 7-h EC. Tear PMNs from 7-h EC at night exhibited hyper-segmented nuclei and more NETosis compared to 2 h EC night, indicating an aged and activated phenotype. The diurnal-nocturnal recruitment pattern of tear PMNs may be driven by increased IL-8 in nighttime tears. Higher degranulation and NETs point to the significant activation of tear PMNs on the ocular surface during prolonged eye closure at night.

## Introduction

Neutrophils (polymorphonuclear neutrophils, PMNs) are the first white blood cells to arrive upon tissue infection or damage and can defend against pathogens through several functional activities, such as oxidative burst, degranulation, and release of neutrophil extracellular traps (NETs)^1^. Previous studies showed that PMNs are recruited to peripheral tissues, such as the mouth^2^, the lungs^3^, and the ocular surface^4^ under healthy conditions (i.e., in the absence of an inflammatory reaction). While the eye is considered an immune privilege site, a large number of leukocytes, with the main population being PMNs^5^, can be collected from the ocular surface non-invasively using a gentle eye wash on waking, following prolonged eye closure^4–9^. Only a few tear PMNs have been collected during the daytime^10,11^, suggesting that infiltration of PMNs to the ocular surface may follow the circadian rhythm, although hypoxia caused by prolonged eye closure may also play a role in leukocyte recruitment^12^.

The oscillations in physiological and behavioural activities enable light-sensitive organisms to anticipate and adapt to environmental changes, such as variations in light exposure and food, which creates the circadian rhythm in an approximately 24-h cycle^13^. Depending on cell types, rhythmic changes in immune cell numbers in the circulation exist; for example, naïve CD4^+^ and CD8^+^ T lymphocytes peak at night^14,15^, while natural killer cells^16^ and effector T cells^15^ peak during the day. Additionally, the circadian infiltration of immune cells to peripheral tissues has been reported in several studies^17–20^. The number of lymphocytes homing to lymph nodes peaks at night^21^, while the increased number of PMNs are recruited to the lungs^22^ and the kidney^23^. Furthermore, the concentrations of proinflammatory mediators, such as interleukin-2 (IL-2), IL-6, IL-12, and tumour necrosis factor-alpha, have been reported to reach their maximum at night, which may play a role in attracting immune cells to the sites^24^.

The guidance of PMNs by chemoattractants, such as complement anaphylatoxin C5a, IL-8, and leukotriene B_4_ (LTB_4_), to local sites, is known as chemotaxis. PMNs sense and follow the concentration gradient through the interaction of chemoattractants and their G-coupled protein receptors (GPCR) expressed on the cell surface, which induces the subsequent transmigration cascade in blood-circulating PMNs including rolling, arresting, crawling, and eventually migrating to the tissues^25^. Thakur et al. reported an increase in IL-8 and LTB_4_ concentrations in the closed-eye tears with increasing eye closure time^26^. Additionally, Mahajan et al. showed that C5a level peaked at awakening^8^. Taken together, such findings may suggest that the recruitment of tear PMNs to the closed-eye environment may be driven by the presence of chemoattractants in tears. However, research is needed to confirm the association between the recruitment of tear PMNs and chemoattractants across the day.

The high numbers of tear PMNs collected from healthy eyes upon waking are quiescent, exhibiting limited activation/pro-inflammatory response upon stimulation with N-formyl-methionyl-leucyl-phenylalanine (fMLP), ﻿lipopolysaccharide (LPS), IL-8, calcium ionophore, and phorbol-12-myristate-13-acetate (PMA) in vitro^4,6,9^*.* It has thus been hypothesized that tear PMNs may have undergone activation while in the closed-eye environment, and become unresponsive to additional stimulation. NETs have gathered significant interest recently as a sign of neutrophil activation and are DNA filaments decorated with granular proteins, such as myeloperoxidase, neutrophil elastase, and lactoferrin that have been expelled from the neutrophils^27,28^. The presence of NETs found in morning eye “discharge”^8^ and upregulation of degranulation markers on tear PMNs^4^ support the concept of prior activation on the ocular surface during sleep (or closed eye conditions). Furthermore, Jin et al. reported a constitutive release of reactive oxygen species (ROS) of tear PMNs collected after nocturnal sleep^5^. This spontaneous ROS release has also been reported in unstimulated aging blood PMNs^29^, which may suggest that tear PMNs are aged when collected after a nighttime sleep.

While recent studies have provided some insights on the phenotype of tear PMNs collected after sleep (prolonged eye closure at night) and potential activation, there is still limited knowledge on the difference between nighttime and daytime and the potential role of chemoattractants. Comparing the difference in cell population across time, the number of tear PMNs and change of phenotypes over time may provide further insights into the mechanisms of recruitment to the ocular surface and the role that tear PMNs play in patrolling and maintaining ocular homeostasis in the anterior segment of the eye. Using a gentle eyewash method to collect cells from the ocular surface, the objectives of this study were thus to investigate the rhythmic infiltration of tear PMNs to the ocular surface and assess their phenotype, in terms of degranulation and maturation state, across different time points. These time points were after 2-h and 7-h of sleep (closed-eye environment) at night, after 2-h sleep (closed-eye environment) during mid-day, and towards the end of the day (open-eye, around 5 pm).

## Materials and methods

### Reagent and monoclonal antibodies

Fluorescein isothiocyanate (FITC)-conjugated antibodies against human CD11b (clone: ICRF44), CD62L, CD15, and CD66b (clone: G10F5), R-phycoerythrin (PE)-conjugated antibodies against human CD54 (clone: LB-2), CD184, and CD63 (clone: H5C6), and R-phycoerythrin- cytochrome 5 (PE-Cy5)-conjugated antibodies against CD45 (clone: HI30) and CD15 (clone: HI98) were purchased from Becton Dickinson Pharmingen (San Diego, California, USA). IL-8 ELISA kit, FITC-conjugated anti-mouse IgG F(ab’)2 CF 488A, May-Grünwald’s stain, DNase I, and fMLP were from Sigma-Aldrich Co. (Oakville, Ontario, Canada). FITC-conjugated antibody against myeloperoxidase, anti-histone H3 (citrulline R2 + R8 + R17) antibody, PE-conjugated anti-goat IgG, and C5a ELISA were purchased from Abcam (Waltham, Massachusetts, USA). Anti-lactoferrin antibody was purchased from Cedarlane (Burlington, Ontario, Canada). Anti-hCXCR1/IL8 RA antibody was purchased from R&D systems (Minneapolis, Minnesota, USA). Phosphate-buffered saline (PBS) was purchased from Lonza (Allendale, New Jersey, USA). Dulbecco’s modified eagle media (DMEM) was purchased from Life Technologies (Burlington, Ontario, Canada).

### Subjects

This study was conducted in accordance with the tenets of the Declaration of Helsinki and received ethics clearance from the University of Waterloo Human Research Ethics Committee (#43743; Waterloo, ON, Canada). Informed consent was obtained from the subjects after explanation of the nature and possible consequences of the study. A total of 20 non-contact lens wearers who were free of any ocular diseases were recruited, consisting of 10 males and 10 females, between the ages of 20 and 36 years of age (28 ± 4). After flow cytometry protocol optimization, an additional 6 participants were recruited to assess the expression of NETs, IL-8Rs, and lactoferrin on tear PMNs collected at different time points.

### Collection of tear PMNs

Each participant was trained to collect cells using the gentle eyewash method previously described by Gorbet et al.^4^ Briefly, participants released saline solution onto their ocular surface using a sterile dropper placed at the inner angle of the eye. The run-off was collected into a sterile 50 mL polypropylene tube placed at the outer angle of the eye.

There were four different collection time points on four different days: three time points of prolonged eye closure (EC) after a full night of sleep (7 h EC night samples), after 2 h of sleep at night (2 h EC night samples), after 2 h of eye closure or sleep in the afternoon (2 h EC day samples); and one open-eye day collection towards the end of the day at around 5 pm (end of day samples). The former two, full night and 2 h night, were considered nighttime collections, which were undertaken on two consecutive days, and the latter two, 2 h day and end of the day, were considered daytime collections. Participants collected the 7 h EC night samples upon waking in the morning after a full night of sleep (average 7 h), which were delivered to the lab within 2 h. For 2 h EC night samples, participants collected the cells after the 2-h sleep at night around 2 am, which were delivered to the lab in the early morning. Participants collected the 2 h EC day samples after closing their eyes for 2 h in the afternoon (after 1 pm). For the end of day samples, participants collected the cells from open eyes in the late afternoon around 5 pm. All daytime collections were delivered to the lab within 2 h of collection.

All collected eyewash samples were centrifuged at 280 × g for 5 min. The supernatants were kept for protein analysis, and the cell pellets were resuspended in PBS. Cell counts were performed using the MOXI Z automated cell counter (ORFLO, Hailey, Idaho, USA), gating on PMN population based on cell diameter, between 6.5 and 9 mm.

### Chemokine analysis

For nighttime collection samples, 1 mL of the eyewash supernatant was transferred to 1.5 mL Eppendorf tubes and stored at – 80 °C for future ELISA analysis. The supernatants of daytime collection samples were transferred to Amicon Ultra-15 centrifugal concentrators with 10,000 Dalton molecular weight cutoff filters (Sigma, Ontario, Canada), followed by centrifugation at 3500×*g* for 20 min. The concentrates were transferred to 1.5 mL Eppendorf tubes and stored at – 80 °C until processing. The concentrations of IL-8 and complement C5a were measured using ELISA, and final concentrations were corrected with the initial volume of eyewashes returned by participants.

### Cell histological staining

After incubation, cells were centrifuged onto a microscope glass slide using the StatSpin Cytofuge (HemoCue America, Brea, California, USA), at 1000×*g* for 2 min, followed by staining with May-Grünwald’s stain and visualization using an Axiovert microscope (Nikon Instruments Inc., Melville, New York, USA). Pictures of the entire circular area of the cytospin, where the cells deposited, were taken at 10 × magnification using an MS-2000-XY mechanical stage (Applied Scientific Instrument, Oregon, USA) and stitched using NIS elements-Advanced Research software package (Nikon Instruments Inc., Melville, New York, USA). The numbers of PMNs, corneal epithelial cells, and aggregates were then counted.

### Cell activation and immunostaining

Due to the limited cell numbers obtained in the daytime collection, only nighttime collection samples were analysed. Cells were either stimulated with 1.5 µM fMLP for 15 min in the cell incubator (37 °C, 5% CO_2_) or not.

Cells were stained with FITC-CD66b (a marker for specific granules), PE-CD63 (a marker for azurophilic granules), FITC-CD62L (L-selectin), PE-CD184 (a marker for cell maturation state), and PE-Cy-5-CD45 (pan leukocyte antibody) for 20 min in the dark at room temperature. Then, samples were diluted with DMEM/10% fetal bovine serum (FBS) and were ready for flow cytometry analysis.

To assess the degranulation of specific granules and NETs release, cells were stained with anti-lactoferrin antibody for 30 min at room temperature in the dark. The cells were subsequently washed with DMEM/10% FBS at 280×*g* for 5 min, followed by staining with FITC-conjugated secondary antibody and PE-Cy5-CD45 for 30 min at room temperature in the dark. Cells were washed at 280×*g* for 5 min and were ready for flow cytometry analysis.

To identify NETs, samples were stained with anti-citrullinated-histones (H_3_Cit) antibody for 30 min at room temperature in the dark, followed by the staining with PE-conjugated secondary antibody, FITC-anti-myeloperoxidase (MPO) antibody, and PE-Cy-5-CD15 for another 30 min at room temperature in the dark. Samples were diluted in DMEM/10%FBS and immediately analysed by flow cytometry.

### Flow cytometry

The PMN population was identified based on double gating the cells that were high in side-scattered light (granularity), low in forward-scatter light (size), and CD45 + using a Becton Dickinson FACS Calibur flow cytometer (Mountain View, California, USA) and associated CELLQuest Software. ﻿At least 2000 PMN events were acquired and mean fluorescent intensities (MFI) were recorded for all samples.

The release of NETs by tear PMNs was also analyzed through flow cytometry following a previously published protocol for NETs^30^, and CD15 was used to identify PMNs. Cells were not permeabilized and fixed, hence the surface expressions of MPO, and H3Cit can be used to quantify the presence of NETs on tear PMNs.

### Statistics

Results are presented as mean ± standard deviation. The paired t-test if normally distributed and Wilcoxon signed-rank test if not normally distributed were used to compute statistical significance. Correlations were evaluated through the Spearman correlation test. A p-value of less than 0.05 was required for statistical significance, and all the statistical analysis was performed via IBM SPSS Software (IBM Canada Ltd., Markham, Ontario, Canada). All figures were plotted using GraphPad Prism Software (GraphPad Software, San Diego, California, USA). Outliers were identified by calculating the interquartile range and were removed.

## Results

### Cell count and nuclei shapes of tear PMNs across different time points

**Figure 1:**
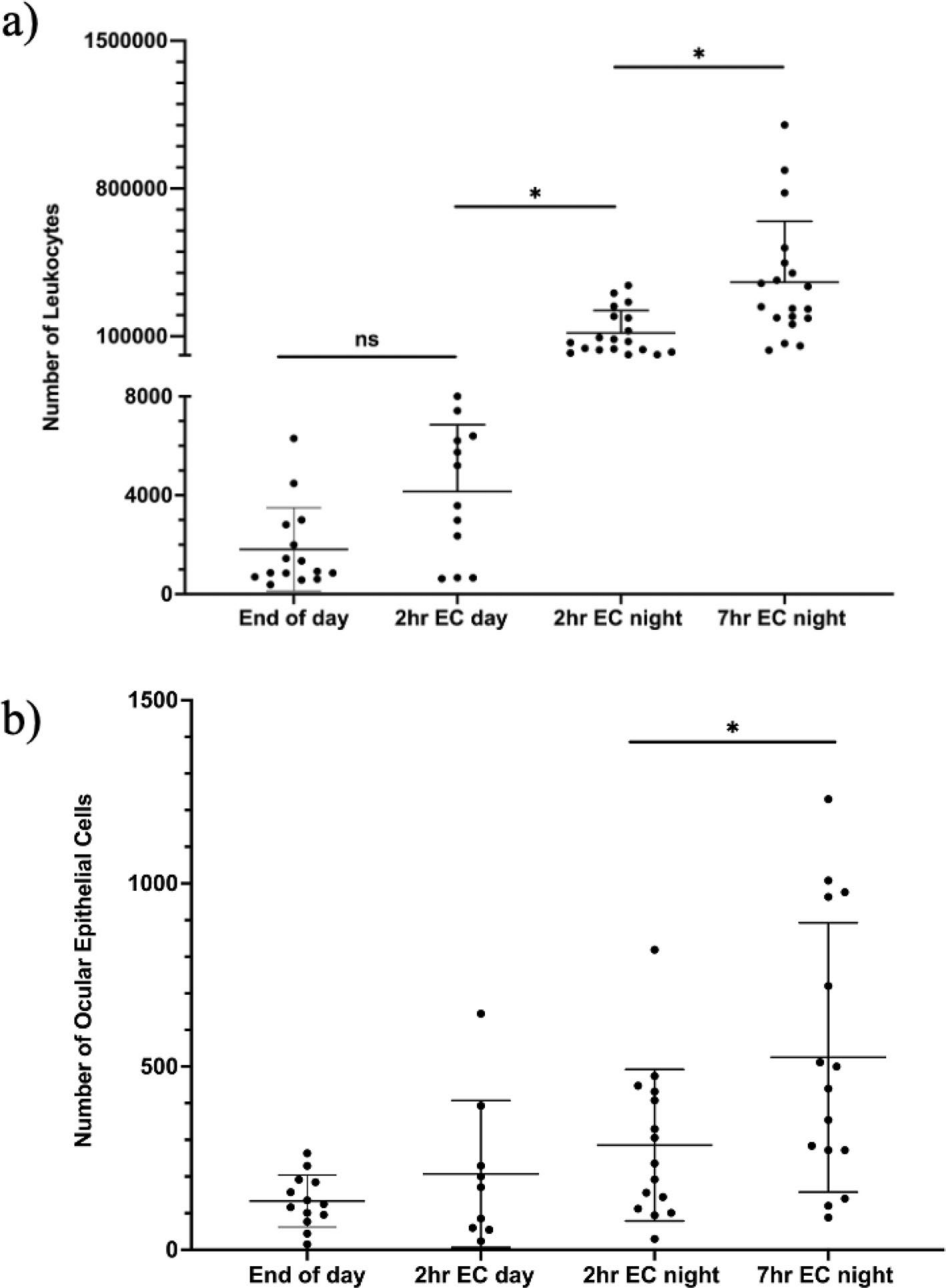
Number of cells collected from the ocular surface at different time points. (**a**) Number of leukocytes was determined using a MOXI-Z automated cell counter. (**b**) Number of ocular epithelial cells from cytospin. Values are presented as means ± standard deviations, n = 12–19 for PMNs, and n = 9–15 for ocular epithelial cells. Wilcoxon matched-pairs signed rank test and paired t-test were conducted to compare the paired data values between end of day and 2 h EC day, 2 h EC day and 2 h EC night, and 2 h EC night and 7 h EC night, *ns* not statistically significant, *significantly different, *p* < 0.006. EC: eye closure.

As reported in Fig. 1a, the increased presence of tear leukocytes in the eyewash depended on time of collection and length of eye closure. The average number of leukocytes collected after 7 h of eye closure at night (full night sleep) was 3.3 × 10^5^, the highest count among all time points. Significantly fewer leukocytes were collected after 2 h of eye closure at night, with an average cell count of 1.4 × 10^5^ (*p* = 0.005), suggesting an increase in leukocyte recruitment with longer eye closure time at night. The numbers of leukocytes present in the nighttime collections were significantly higher than the cell counts in either daytime collection, with daytime collections yielding fewer than 3000 leukocytes on average. Significantly fewer leukocytes were also collected after 2 h of eye closure during the day when compared to the 2 h of eye closure at nighttime (p < 0.001), highlighting that eye closure alone was not driving leukocyte presence on the ocular surface.

A small number of ocular epithelial cells (less than a thousand on average) were also present in the eyewash and counted from the cytospin (see Fig. 1b). The highest number of ocular epithelial cells in the eyewash were observed after a full night of sleep (*p* < 0.006).

**Figure 2:**
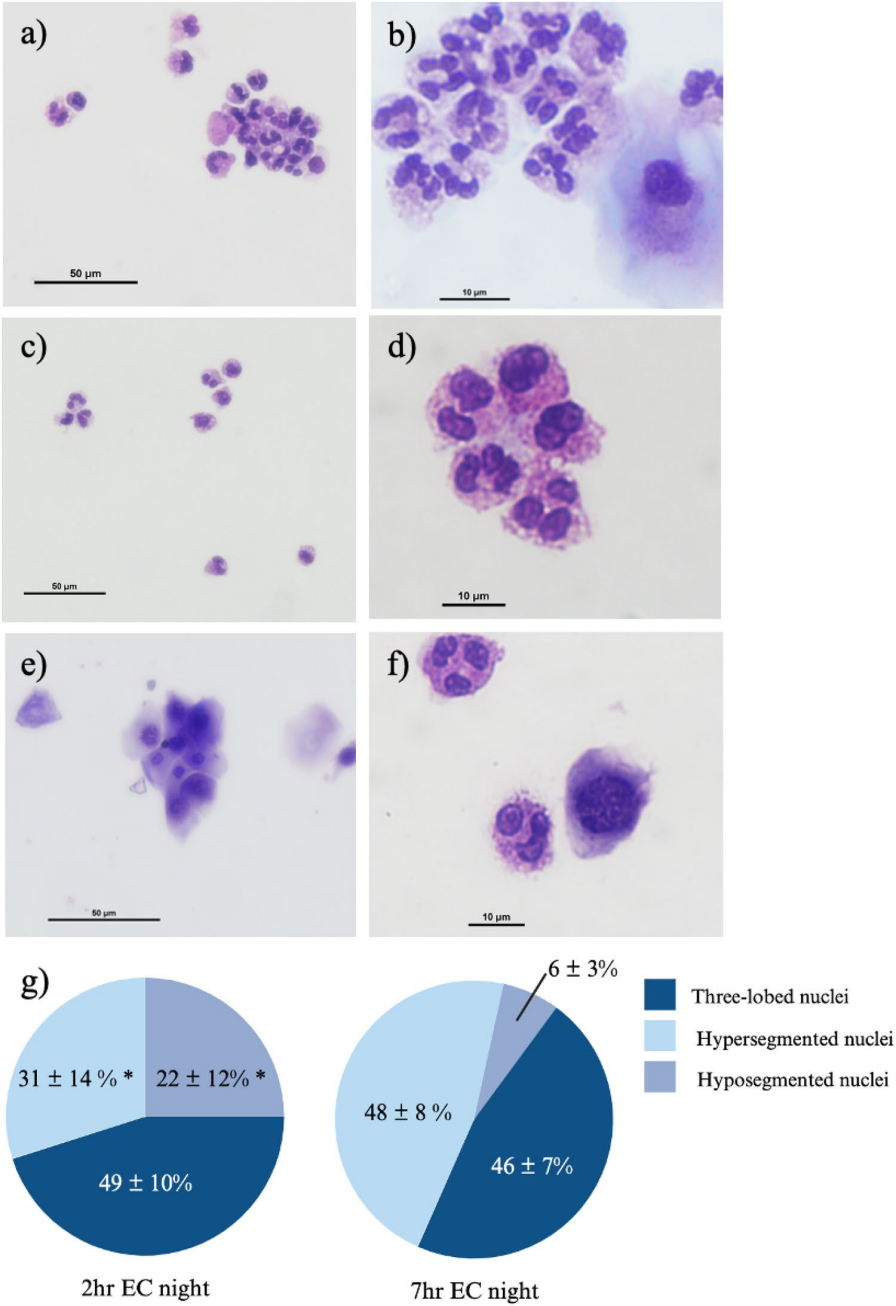
Analysis of the nucleus morphology of tear PMNs collected after a full night of sleep (**a**,**b**), after 2 h sleep at night (**c**,**d**), and during the day (**e**,**f**). Note that in (**e**), only cluster of ocular epithelial cells can be observed as little to no PMNs are collected during daytime. The percentage of 2 h EC night and 7 h EC night tear PMNs exhibiting three-lobed, hyper-segmented, and hypo-segmented nuclei, n = 10–16, *significantly different from 7 h EC night tear PMNs, *p* ≤ 0.024 (**g**).

One of the hallmarks of fresh blood PMNs is their multi-lobed nuclei (2–4 lobules)^31^, and nuclear morphology can provide information on cell maturation state. On the cytospins, hyper-segmented nuclei (more than 4 lobes of nuclei) were commonly observed in the 7 h EC night tear PMNs (Fig. 2a-b), while some 2 h EC night tear PMNs appeared to exhibit hypo-segmented nuclei (Fig. 2c-d). From the daytime collection, ocular surface epithelial cells were predominantly present in the eyewash (Fig. 2e), and when present, the tear PMNs harvested displayed normal (tri-lobar) nuclear morphology (Fig. 2f), similar to fresh circulating blood PMNs. As seen in Fig. 2g, a significantly higher percentage (23%) of the 2 h EC night tear PMNs exhibited hypo-segmented nuclei compared to the 7 h EC night tear PMNs (*p* = 0.005). Conversely, a significantly higher percentage (48%) of the 7 h EC night tear PMNs showed hyper-segmented nuclei compared to the 2 h EC night tear PMNs (*p* = 0.024). As hyper-segmentation has been associated with aging and maturation, to further confirm our histological observations, the expression of CD184, a marker for cell aging^32^, was characterized on tear PMNs by flow cytometry. A significantly higher expression of CD184 was observed on 7 h EC night tear PMNs compared to 2 h EC night tear PMNs, 62 ± 14 and 46 ± 12 (Mean Fluorescence Intensity) respectively (*p* = 0.008), suggesting that tear PMNs collected after a full night of sleep were more aged than 2 h EC night tear PMNs, and corroborating the microscopy observations around nuclear morphology.

### The correlation between proinflammatory mediators and tear PMN numbers

IL-8 and C5a have been shown to be present in the tear film and are chemoattractants, recruiting leukocytes to tissues^1^. The average concentration of IL-8 in 7 h EC night samples was significantly higher than in the 2 h EC night samples, as shown in Table 1 (*p* < 0.001). IL-8 concentration in the 2 h eye closure at night eyewash was significantly higher than in the eyewash collected after 2 h eye closure during the day (*p* = 0.001), suggesting that the increase in IL-8 concentration in 2 h EC night compared to 2 h EC day samples was unlikely due to simple “accumulation” of IL-8 in the closed-eye environment and highlighting the pro-inflammatory environment of the nocturnal closed-eye environment. No difference in IL-8 concentrations in the eyewash was observed between the two daytime collection samples.

**Table 1:** Eyewash IL-8 and C5a concentrations collected at different time points.

Collection time	IL-8 concentration (pg/mL)	C5a concentration (pg/mL)
End of day	29 ± 20	4 ± 10
2 h EC day	33 ± 21	11 ± 22
2 h EC night	185 ± 162^#^	96 ± 191^#^
7 h EC night	669 ± 412*	176 ± 176*

Values are presented as means ± standard deviations, IL-8 concentration: n = 12–17; C5a concentration: n = 9–14. Wilcoxon matched-pairs signed rank test was conducted to compare the paired data values between end of day and 2 h EC day, 2 h EC day and 2 h EC night, and 2 h EC night and 7 h EC night.

*EC* eye closure.

*Significantly different from 2 h EC night, *p* ≤ 0.005.

^#^Significantly different from 2 h EC day, *p* < 0.04.

C5a concentration significantly increased between 2 and 7 h EC night samples (*p* = 0.005), whereas C5a was barely detected in the daytime collection samples. Furthermore, the concentration of C5a in the 2 h EC night samples was significantly higher than the 2 h EC day samples (*p* = 0.031).

The changes in IL-8 concentrations across different time points followed a similar trend to the PMN count. The number of leukocytes collected from the ocular surface was positively correlated with IL-8 concentration (*p* < 0.014, r = 0.678, see Fig. S1). However, there was no correlation between leukocyte number and C5a concentration in the eyewash (*p* = 0.984, r = 0.003, see Fig. S2).

### PMN phenotypes from 2 and 7 h EC night samples

**Figure 3:**
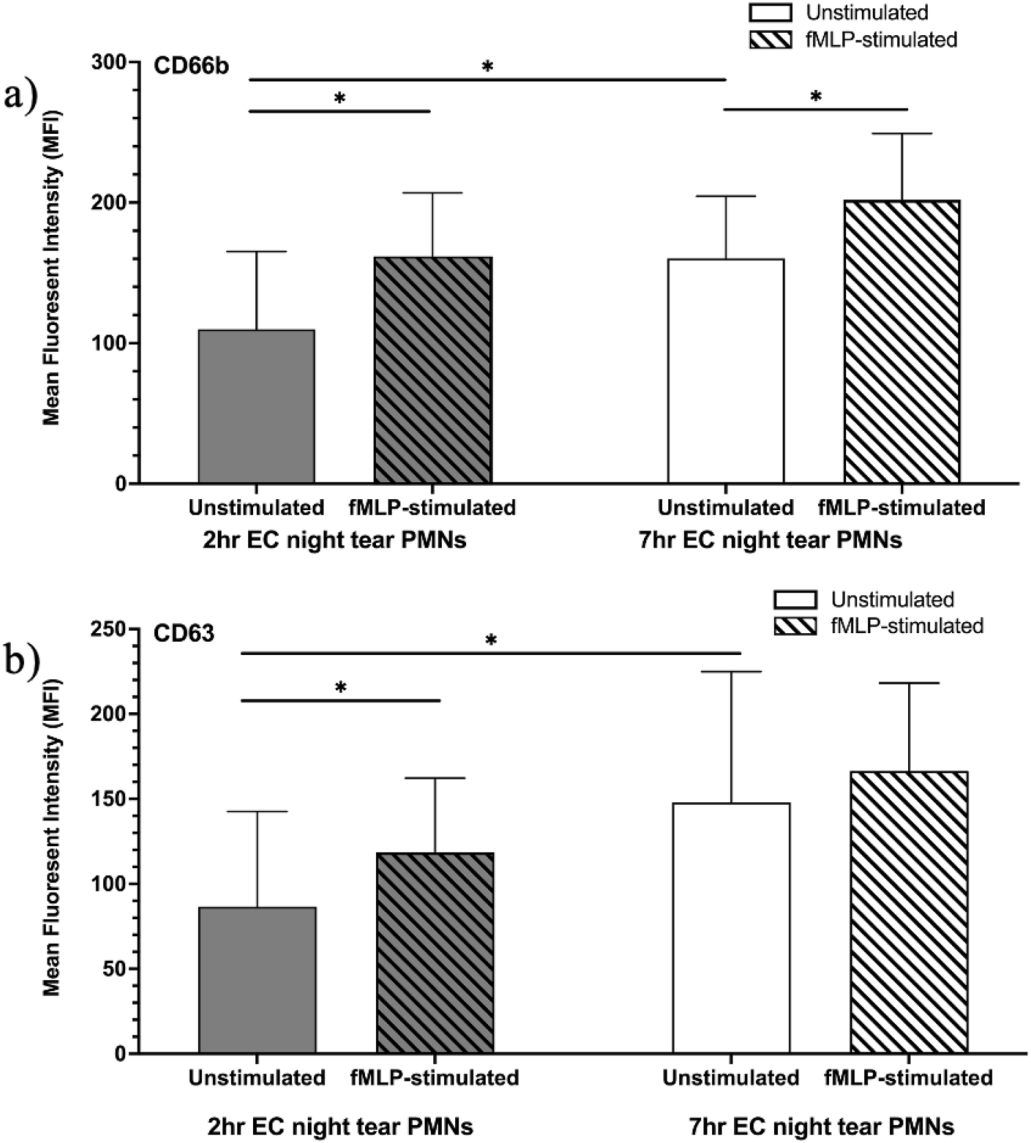
Changes in the expression levels of (**a**) CD66b and (**b**) CD63 on 2 h and 7 h EC night tear PMNs under unstimulated and fMLP-stimulated conditions. Tear PMNs were stained with CD63 and CD66b, and samples were analyzed by flow cytometry. Results are presented as mean ± standard deviations, n = 13–20. *EC* eye closure, *ns* not statistically significant, *significantly different (*p* ≤ 0.012).

Due to the low PMN numbers from the daytime collections, with few cells remaining after cell count and cytology, only the phenotypes of 7 h EC and 2 h EC night tear PMNs were evaluated. Tear PMNs collected after a full night of sleep (7 h EC) exhibited a significantly higher expression of CD63 (*p* = 0.007) and CD66b (*p* = 0.012) compared to 2 h EC night tear PMNs (Fig. 3a,b, respectively).

Upon stimulation with fMLP, 2 h EC night tear PMNs also demonstrated higher levels of CD66b (1.61 ± 0.35 vs. 1.27 ± 0.18, p = 0.001) and CD63 (1.87 ± 0.56 vs. 1.32 ± 0.33, *p* = 0.002) expressions compared to 7 h EC night tear PMNs, further highlighting changes in degranulation and activation potential associated with residing for longer period in the closed-eye environment and leading to lower granule content.

The levels of expression of CD62L, CD11b and CD54, which have been mostly associated with transmigration phenotype^33,34^, were similar on the 2 h and 7 h night tear PMNs (see Table S1).

While results did not reach statistical significance, noteworthy is the fact that CD66b expression on 2 h night tear PMNs collected from female participants was higher than that of males, with values of 143 ± 57 and 117 ± 65, respectively. Additionally, the activation ratio of 2 h night tear PMNs in females (1.39 ± 0.32) was found to be lower than that of males (1.74 vs. 0.37).

Furthermore, 7 h EC night tear PMNs showed a significantly higher level of lactoferrin (*p* = 0.007) and lower expression of IL-8Rs than 2 h EC night tear PMNs (*p* = 0.002), as shown in Table 2, indicating the increased expression of lactoferrin and internalization of IL-8Rs are associated with the phenotype of activated PMNs^35^.

**Table 2:** The mean fluorescent values of lactoferrin expression on 7 h EC and 2 h EC night tear PMNs.

		2 h EC night tear PMNs	7 h EC night tear PMNs
Lactoferrin	Unstimulated	120 ± 82	285 ± 107*
	fMLP stimulated	173 ± 105	324 ± 200
	Ratio of stimulated vs unstimulated	1.4 ± 0.4	1.2 ± 0.3
IL-8Rs	Unstimulated	25 ± 7	17 ± 4*

Tear PMNs were stained with lactoferrin, and immediately characterized by flow cytometry. Results are presented as mean ± standard deviations. n = 6.

*Significantly different from the 2 h night PMNs (*p* < 0.007).

### Neutrophil extracellular traps (NETs)

**Figure 4:**
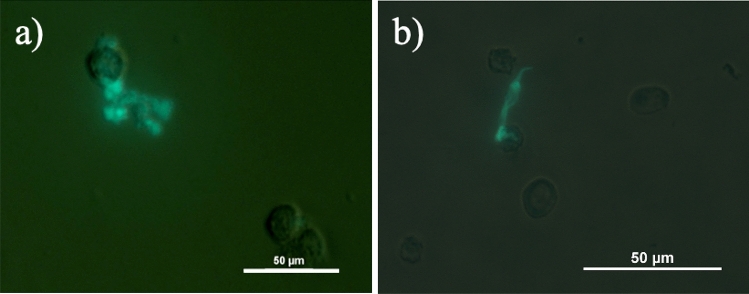
Tear PMNs exhibiting NETs in tear samples collected at night, stained with antibodies against lactoferrin and its FITC-conjugated secondary antibody.

**Figure 5:**
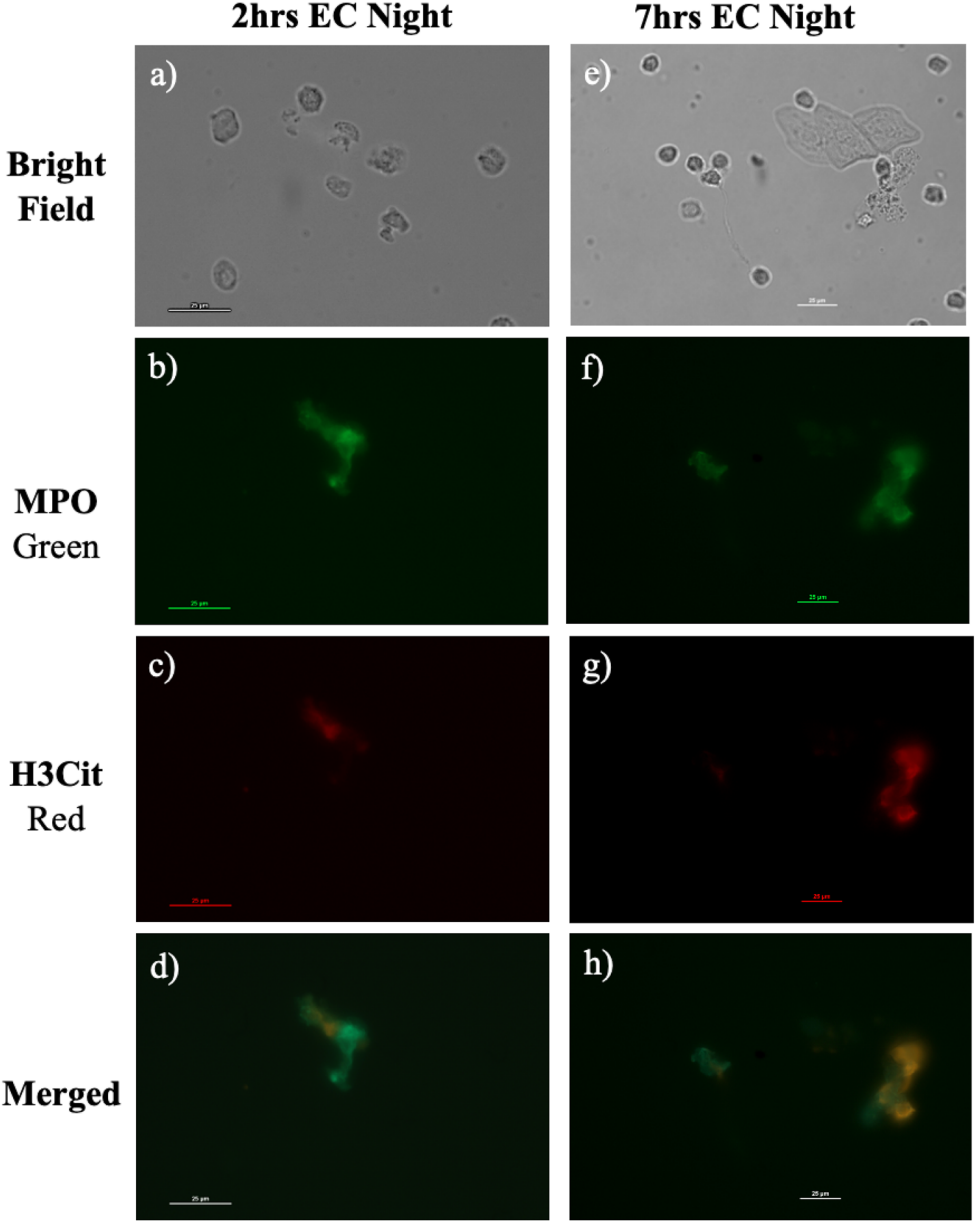
Tear PMNs exhibiting NETs in tear samples collected after 2 h (**a**–**d**) and 7 h (e–h) EC night, stained with FITC-conjugated MPO and H3Cit using a PE-conjugated secondary antibody. (**a**,**e**) bright field; (**b**,**f**) green channel showing FITC-conjugated anti-myeloperoxidase (MPO) antibodies; (**c**,**g**) red channel displaying anti-citrullinated histones (H3Cit) antibodies with its PE-conjugated secondary antibody; (**d**,**h**) colocalization of MPO and H3Cit (orange).

As could be observed in Fig. 2a, tear PMNs collected from a full night of sleep appeared to form aggregates/clusters on the cytospin. Upon treatment with DNase I, a 70% reduction in the number of aggregates was observed (see Fig. S3), suggesting the presence of NETs on tear PMNs. NETs on PMNs were visualized by fluorescent microscopy with lactoferrin (Fig. 4), myeloperoxidase (MPO), and citrullinated histones (H3Cit) (Fig. 5)^28,36^. NETs were then further quantified by flow cytometry with MPO and H3Cit^37^. As shown in Table 3, tear PMNs collected after a full night of sleep showed significantly higher levels of MPO and H3Cit (*p* = 0.046). Compared to 2 h EC night tear PMNs, significantly more 7 h EC night tear PMNs exhibited NETs, staining positive for both MPO and H3Cit, 26% ± 14% vs. 11% ± 7% (*p* = 0.043) (see supplementary Fig. S5).

**Table 3:** Changes in the levels of MPO, and H3Cit on 2 h and 7 h EC night tear PMNs.

	Mean fluorescent intensity (MFI)	
	2 h EC night	7 h EC night
MPO	13 ± 2	21 ± 10*
H3Cit	9 ± 4	13 ± 7*

Tear PMNs were stained with MPO and H3Cit, and immediately characterized by flow cytometry. Results are presented as mean ± standard deviations. n = 6.

*Significantly different from the 2 h night PMNs (*p* < 0.05).

## Discussion

Two hypotheses have been proposed regarding the origin of tear PMNs, whereby they arise from the lacrimal glands and are washed away due to blinking during the day and then accumulate at night during prolonged eye closure^38^, or leukocytes extravasate from conjunctival blood vessels due to the presence of chemoattractants^4^. The fact that very few leukocytes were observed following 2 h eye closure during the day, while over 100,000 leukocytes were collected after 2 h eye closure at night strongly supports the hypothesis that tear PMNs and other leukocytes found on the ocular surface during sleep/eye closure extravasate from the blood vessels in response to a chemoattractant. Noteworthy is that significantly higher numbers of leukocytes were observed after 7 h of eye closure compared to 2 h of eye closure at night, suggesting that there is also continuous extravasation of leukocytes, which is likely related to the continuous presence of chemoattractants, such as IL-8 and C5a.

An increase in IL-8 concentration with increasing eye closure time during the night was observed in this study, corroborating results from tear collections (using capillary tubes)^26^, and indicating that the eyewash method provides an effective way to sample the closed eye environment, whereby both tear proteins and cells can be assessed reliably. Chemoattractants present in tears have the capability to attract PMNs to the ocular surface, as blood-isolated PMNs showed enhanced chemotaxis in the presence of closed-eye tears collected after sleep compared to open-eye tears^26^. The positive correlation between tear PMN number and IL-8 concentration but not with C5a concentration indicates that IL-8 may contribute significantly to the recruitment of tear PMNs to the ocular surface.

Our study provides the first evidence of a difference in nuclear morphology in tear PMNs. The distribution in nuclear morphology of 2 h closed-eye night tear PMNs was found to be similar to that previously reported for a normal/healthy blood neutrophil population^39^. In comparison, a significantly higher percentage of hyper-segmented PMNs were observed in the 7 h closed-eye night tear PMNs, a nuclear morphology that has been suggested to be indicative of cell aging and activation^19,31,40^. The higher presence of hyper-segmented PMNs is in line with our observations indicating a high level of activation of tear PMNs collected upon awaking (high degranulation, increased presence of NETs and higher levels of CD184). Additionally, the limited degranulation response of 7 h EC night tear PMNs to fMLP stimulation points towards the aging mechanisms and lower granular content due to prior activation, similar to aged blood PMNs reported to have a lower granule content and NET-forming capability compared to young or fresh PMNs^41^.

Several research groups, including our own, have hypothesized through indirect evidence that tear PMNs collected after a full night of sleep are being activated when they are in the nocturnal closed-eye environment^4,5,8,9^. In the current study, 7 h EC night tear PMNs exhibited higher levels of degranulation markers (CD66b, CD63 and lactoferrin) and NETs markers (H3Cit and MPO) and lower levels of IL-8Rs compared to the 2 h EC night tear PMNs, providing direct evidence of activation/degranulation of tear PMNs in the nighttime closed-eye environment over prolonged residence time. While our study focussed on receptor expression and NETs, many PMN functions are interconnected and research has also demonstrated the importance of a functional NADPH oxidase in inducing NETosis, as evidenced by impaired NETs release in PMNs from chronic granulomatous disease (CGD) patients and NADPH oxidase-knockout mice^42^. To provide better insights into the activation and regulatory effects of tear PMNs on ocular surface homeostasis and inflammation, further investigation will be needed to correlate NETs with ROS production in tear PMNs. Sex has also been reported to have a significant effect on the inflammatory response^43,44^, and while our results showed that some differences in tear PMNs phenotypes existed between male and female, these did not reach statistical significance. Our prior study on ROS production with a similar sample size had reached similar conclusions^5^. Further investigations with larger sample sizes will be needed to confirm the effects of sex on tear PMNs activation in the closed eye environment. Overall, our findings indicate that night-infiltrating tear PMNs become activated as they interact with the ocular surface environment during nighttime closed-eye conditions, potentially to eliminate pathogens and clean debris accumulated on the ocular surface throughout the day, ultimately enhancing the eye’s defense mechanisms and contributing to ocular surface homeostasis. Given the double-edged role of neutrophil functions and the susceptibility of the eye to immune-mediated damage, there likely exist mechanisms within the ocular surface environment to protect against degranulation and other inflammatory mediators released by tear PMNs. Extravasated neutrophils have been reported to contribute to homeostasis and diseases in tissues such as the lungs^4^, the mouth^45^ and the uterus^46^. Further research will be needed to clearly identify the role of tear PMNs on ocular surface homeostasis; this may shed light on the development of various ocular surface pathologies and how the presence of a biomaterial may affect the innate immune response on the ocular surface.

Our results point towards the circadian recruitment of tear PMNs to the ocular surface, with the infiltration of tear PMNs peaking at night. Tan et al.^10^ had reported that only a few tear PMNs could be collected during the first 3 h of sleep, which contradicts observations by Postnikoff et al.^6^ and our current study. This inconsistency may be due to differences in cell collection methods, with Tan et al. using capillary tubes, while the other studies used a gentle eyewash method. Tear PMN cell aging also appear to follow a circadian rhythm. Diurnal cell aging has been reported in blood circulating PMNs by Adrover et al.^47^, and may be regulated by their intrinsic molecular clock^47^ or the systemic mechanism^19^. In other tissues, such as in the lungs, PMNs have also been shown to extravasate to the lung tissues during the resting (night) period due to the secretion of CXCL5 produced by pulmonary epithelial cells, which may be controlled by the circadian system (including both the circadian clock and neurohormones) and play an important role in the response to bacterial infection^48^. A study by Alenezi et al. recently highlighted how the circadian rhythm may affect cells of the ocular surface, whereby genes involved in immune defence, mucin production, cell signaling and turnover in bulbar conjunctival cells were found to be differentially upregulated in the early morning compared to the evening^49^. More research will be needed to determine the role of the circadian rhythm on ocular surface homeostasis.

## Conclusion

Our study demonstrated that the infiltration of tear PMNs to the ocular surface in the closed-eye environment peaked at night under healthy conditions, suggesting a circadian rhythm of the recruitment of tear PMNs to the eyes. Tear PMNs undergo significant activation with increasing eye-closure time at night, showing increased degranulation, NETs, and upregulation of CXCR4 markers. The activation and presence of these inflammatory mediators highlight the potential role of neutrophils in ocular surface homeostasis in the closed-eye environment at night. An imbalance in regulation or overproduction of mediators may lead to ocular surface damage and contribute to pathology. Many diseases, such as rheumatoid arthritis^50^ and asthma^51^, have a time-dependent severity of symptoms. Although there is limited research on the variation in symptom severity across the day in ocular complications, our findings suggest that circadian regulation of tear PMNs could play a role in the pathogenesis of ocular conditions, such as dry eye^11,52^ and keratoconus^53^. Further research will be needed to better understand the role of tear PMNs activation in ocular surface inflammation and their interactions with the protective mechanisms of the ocular surface in the nocturnal/diurnal cycle.

### Supplementary Information

Supplementary Information.

## Data Availability

The datasets analysed during the current study are available from the corresponding author upon reasonable request.

## References

[1] Hellewell PG, Williams TJ (1994). Immunopharmacology of Neutrophils.

[2] Rijkschroeff P, Jansen IDC, Van Der Weijden FA (2016). Oral polymorphonuclear neutrophil characteristics in relation to oral health: A cross-sectional, observational clinical study. Int. J. Oral Sci..

[3] Fortunati E, Kazemier KM, Grutters JC, Koenderman L, Van Den Bosch VJMM (2009). Human neutrophils switch to an activated phenotype after homing to the lung irrespective of inflammatory disease. Clin. Exp. Immunol..

[4] Gorbet M, Postnikoff C, Williams S (2015). The noninflammatory phenotype of neutrophils from the closed-eye environment: A flow cytometry analysis of receptor expression. Investig. Opthalmol. Vis. Sci..

[5] Jin Y, Dixon B, Jones L, Gorbet M (2021). The differential reactive oxygen species production of tear neutrophils in response to various stimuli in vitro. Int. J. Mol. Sci..

[6] Postnikoff CK, Nichols KK (2017). Neutrophil and T-cell homeostasis in the closed eye. Invest. Ophthalmol. Vis. Sci..

[7] Nair AP, D’Souza S, Shetty R (2021). Altered ocular surface immune cell profile in patients with dry eye disease. Ocul. Surf..

[8] Mahajan A, Grüneboom A, Petru L (2019). Frontline Science: Aggregated neutrophil extracellular traps prevent inflammation on the neutrophil-rich ocular surface. J. Leukoc. Biol..

[9] Jin Y, Jones L, Gorbet M (2020). Investigation of the response of tear-film neutrophils to interleukin 8 and their sensitivity to centrifugation, fixation, and incubation. Sci. Rep..

[10] Tan KO, Sack RA, Holden BA, Swarbrick HA (1993). Temporal sequence of changes in tear film composition during sleep. Curr. Eye Res..

[11] Postnikoff CK, Carrie H, Gerald M, Kelly KN (2018). Leukocyte distribution in the open eye tears of normal and dry eye subjects. Curr. Eye Res..

[12] Lodge KM, Cowburn AS, Li W, Condliffe AM (2020). The impact of hypoxia on neutrophil degranulation and consequences for the host. Int. J. Mol. Sci..

[13] Halberg F (1959). Physiologic 24-hour periodicity; general and procedural considerations with reference to the adrenal cycle. Int. Z. Vitaminforsch Beih..

[14] Abo T, Kawate T, Itoh K, Kumagai K (1981). Studies on the bioperiodicity of the immune response. I. Circadian rhythms of human T, B, and K cell traffic in the peripheral blood. J. Immunol..

[15] Dimitrov S, Benedict C, Heutling D (2009). Cortisol and epinephrine control opposing circadian rhythms in T cell subsets. Blood.

[16] Dimitrov S, Lange T, Nohroudi K, Born J (2007). Number and function of circulating human antigen presenting cells regulated by sleep. Sleep.

[17] Logan RW, Sarkar DK (2012). Circadian nature of immune function. Mol. Cell Endocrinol..

[18] Scheiermann C, Kunisaki Y, Frenette PS (2013). Circadian control of the immune system. Nat. Rev. Immunol..

[19] Ella K, Csépányi-Kömi R, Káldi K (2016). Circadian regulation of human peripheral neutrophils. Brain Behav. Immun..

[20] Labrecque N, Cermakian N (2015). Circadian clocks in the immune system. J. Biol. Rhythms.

[21] Druzd D, Matveeva O, Ince L (2017). Lymphocyte circadian clocks control lymph node trafficking and adaptive immune responses. Immunity.

[22] Casanova-Acebes M, Nicolás-Ávila JA, Li JL (2018). Neutrophils instruct homeostatic and pathological states in naive tissues. J. Exp. Med..

[23] Aroca-Crevillén A, Adrover JM, Hidalgo A (2020). Circadian features of neutrophil biology. Front. Immunol..

[24] Lange T, Dimitrov S, Born J (2010). Effects of sleep and circadian rhythm on the human immune system. Ann. N. Y. Acad. Sci..

[25] Ley K, Laudanna C, Cybulsky MI, Nourshargh S (2007). Getting to the site of inflammation: The leukocyte adhesion cascade updated. Nat. Rev. Immunol..

[26] Thakur A, Willcox MDP, Stapleton F (1998). The proinflammatory cytokines and arachidonic acid metabolites in human overnight tears: Homeostatic mechanisms. J. Clin. Immunol..

[27] Pleskova SN, Gorshkova EN, Kriukov RN (2018). Dynamics of formation and morphological features of neutrophil extracellular traps formed under the influence of opsonized *Staphylococcus aureus*. J. Mol. Recogn..

[28] Urban CF, Ermet D, Schmid M (2009). Neutrophil extracellular traps contain calprotectin, a cytosolic protein complex involved in host defense against *Candida albicans*. PLoS Pathog..

[29] Xu Y, Loison F, Luo HR (2010). Neutrophil spontaneous death is mediated by down-regulation of autocrine signaling through GPCR, PI3Kγ, ROS, and actin. Proc. Natl. Acad. Sci. U S A.

[30] Gavillet M, Martinod K, Renella R (2015). Flow cytometric assay for direct quantification of neutrophil extracellular traps in blood samples. Am. J. Hematol..

[31] Lokwani R, Wark PAB, Baines KJ, Barker D, Simpson JL (2019). Hypersegmented airway neutrophils and its association with reduced lung function in adults with obstructive airway disease: an exploratory study. BMJ Open.

[32] Kim JH, Podstawka J, Lou Y (2018). Aged polymorphonuclear leukocytes cause fibrotic interstitial lung disease in the absence of regulation by B cells. Nat. Immunol..

[33] Macey MG, McCarthy DA, Vordermeier S, Newland AC, Brown KA (1995). Effects of cell purification methods on CD11b and l-selectin expression as well as the adherence and activation of leucocytes. J. Immunol. Methods.

[34] Yao Y, Matsushima H, Ohtola JA (2015). Neutrophil priming occurs in a sequential manner and can be visualized in living animals by monitoring IL-1β promoter activation. J. Immunol..

[35] Jones SA, Wolf M, Qin S, Mackay CR, Baggiolini M (1996). Different functions for the interleukin 8 receptors (IL-8R) of human neutrophil leukocytes: NADPH oxidase and phospholipase D are activated through IL-8R1 but not IL-8R2. Proc. Natl. Acad. Sci. USA.

[36] Okubo K, Kamiya M, Urano Y (2016). Lactoferrin suppresses neutrophil extracellular traps release in inflammation. EBioMedicine.

[37] Zharkova O, Tay SH, Lee HY (2019). A flow cytometry-based assay for high-throughput detection and quantification of neutrophil extracellular traps in mixed cell populations. Cytometry A.

[38] Gronert K (2010). The grail for healthy ocular inflammation. Exp. Eye Res..

[39] Chan YK, Tsai MH, Huang DC, Zheng ZH, Hung KD (2010). Leukocyte nucleus segmentation and nucleus lobe counting. BMC Bioinform..

[40] Pillay J, Kamp VM, van Hoffen E (2012). A subset of neutrophils in human systemic inflammation inhibits T cell responses through Mac-1. J. Clin. Investig..

[41] Adrover JM, Aroca-Crevillén A, Crainiciuc G (2020). Programmed ‘disarming’ of the neutrophil proteome reduces the magnitude of inflammation. Nat. Immunol..

[42] Parker H, Winterbourn CC (2013). Reactive oxidants and myeloperoxidase and their involvement in neutrophil extracellular traps. Front. Immunol..

[43] Taneja V (2018). Sex hormones determine immune response. Front. Immunol..

[44] Bouman A, Heineman MJ, Faas MM (2005). Sex hormones and the immune response in humans. Hum. Reprod. Update.

[45] Fine N, Hassanpour S, Borenstein A (2016). Distinct oral neutrophil subsets define health and periodontal disease states. J. Dent. Res..

[46] Smith JM, Wira CR, Fanger MW, Shen L (2006). Human fallopian tube neutrophils: A distinct phenotype from blood neutrophils. Am. J. Reprod. Immunol..

[47] Adrover JM, del Fresno C, Crainiciuc G (2019). A neutrophil timer coordinates immune defense and vascular protection. Immunity.

[48] Gibbs J, Ince L, Matthews L (2014). An epithelial circadian clock controls pulmonary inflammation and glucocorticoid action. Nat. Med..

[49] Alenezi H, Ozkan J, Willcox M, Parnell G, Carnt N (2022). Differential gene expression of the healthy conjunctiva during the day. Contact Lens Anterior Eye..

[50] Fagiani F, Di Marino D, Romagnoli A (2022). Molecular regulations of circadian rhythm and implications for physiology and diseases. Signal Transduct. Target Ther..

[51] Beam WR, Weiner DE, Martin RJ (1992). Timing of prednisone and alterations of airways inflammation in nocturnal asthma. Am. Rev. Respir. Dis..

[52] Postnikoff CK, Held K, Viswanath V, Nichols KK (2020). Enhanced closed eye neutrophil degranulation in dry eye disease. Ocul. Surf..

[53] D’Souza S, Nair AP, Sahu GR (2021). Keratoconus patients exhibit a distinct ocular surface immune cell and inflammatory profile. Sci. Rep..

