# Supplementary material for: Investigation of the rhythmic recruitment of tear neutrophils to the ocular surface and their phenotypes
Source: Sci Rep. 2024 Mar 25;14:7061. doi: 10.1038/s41598-024-57311-8 (PMC10963749; doi:10.1038/s41598-024-57311-8)
Supplement: Supplementary file 1 — Supplementary Information. [file 41598_2024_57311_MOESM1_ESM.docx]

**Supplementary Material**


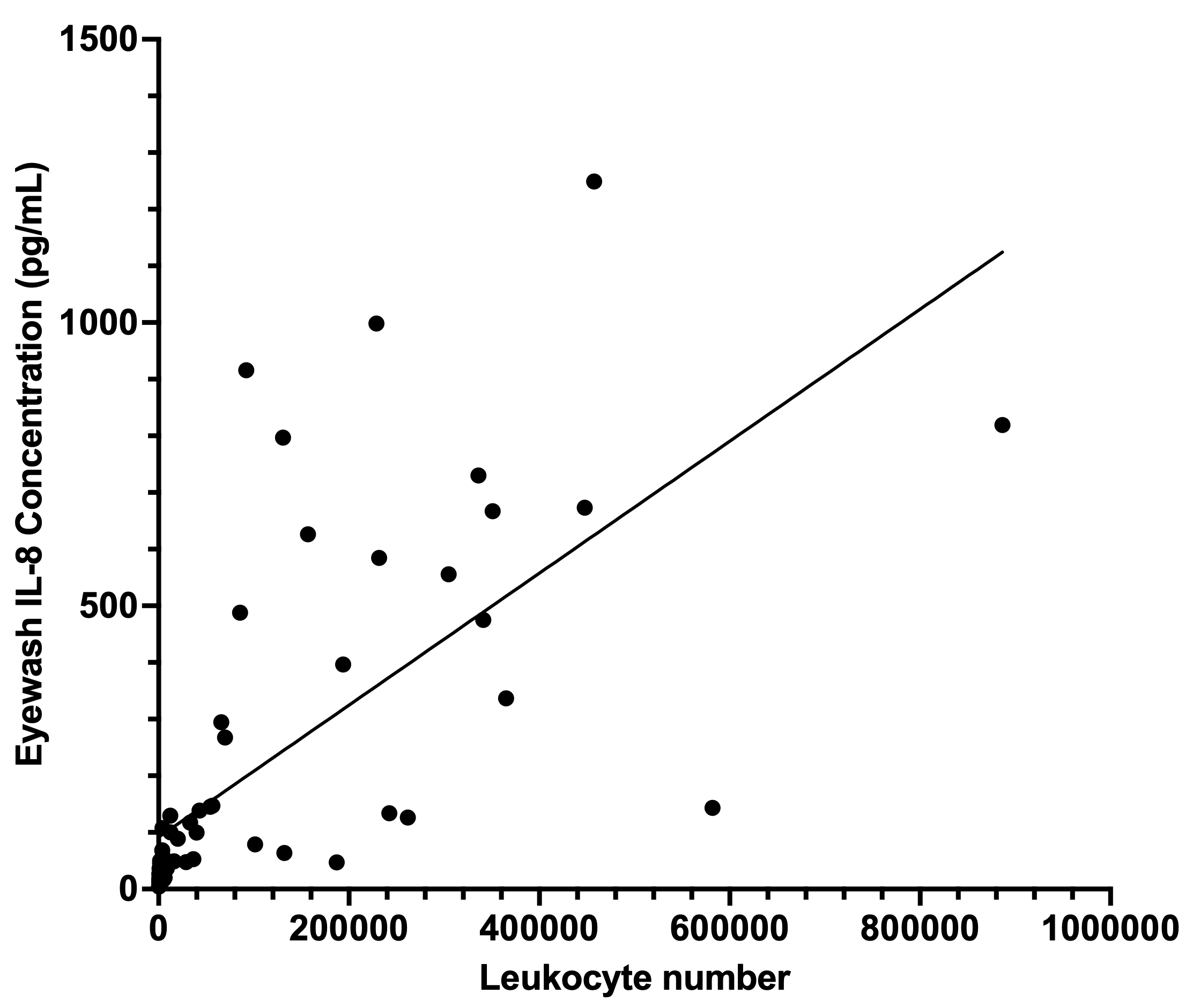


**Figure S1.** Correlation between eyewash IL-8 concentration and leukocyte number. r = 0.675, *p* = 0.001.


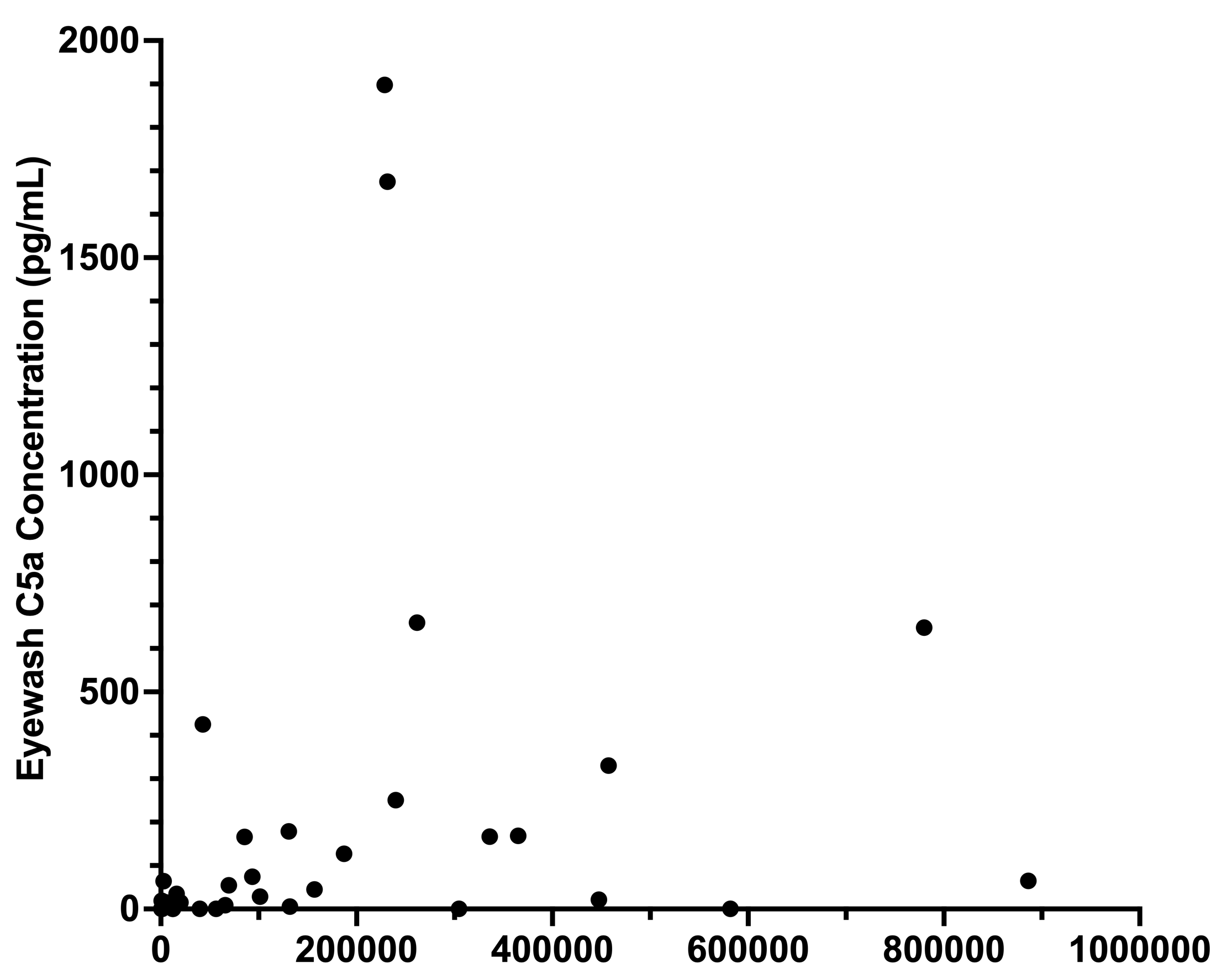


**Figure S2.** Lack of correlation between eyewash C5a concentration and leukocyte number. r = 0.003, *p* = 0.984.


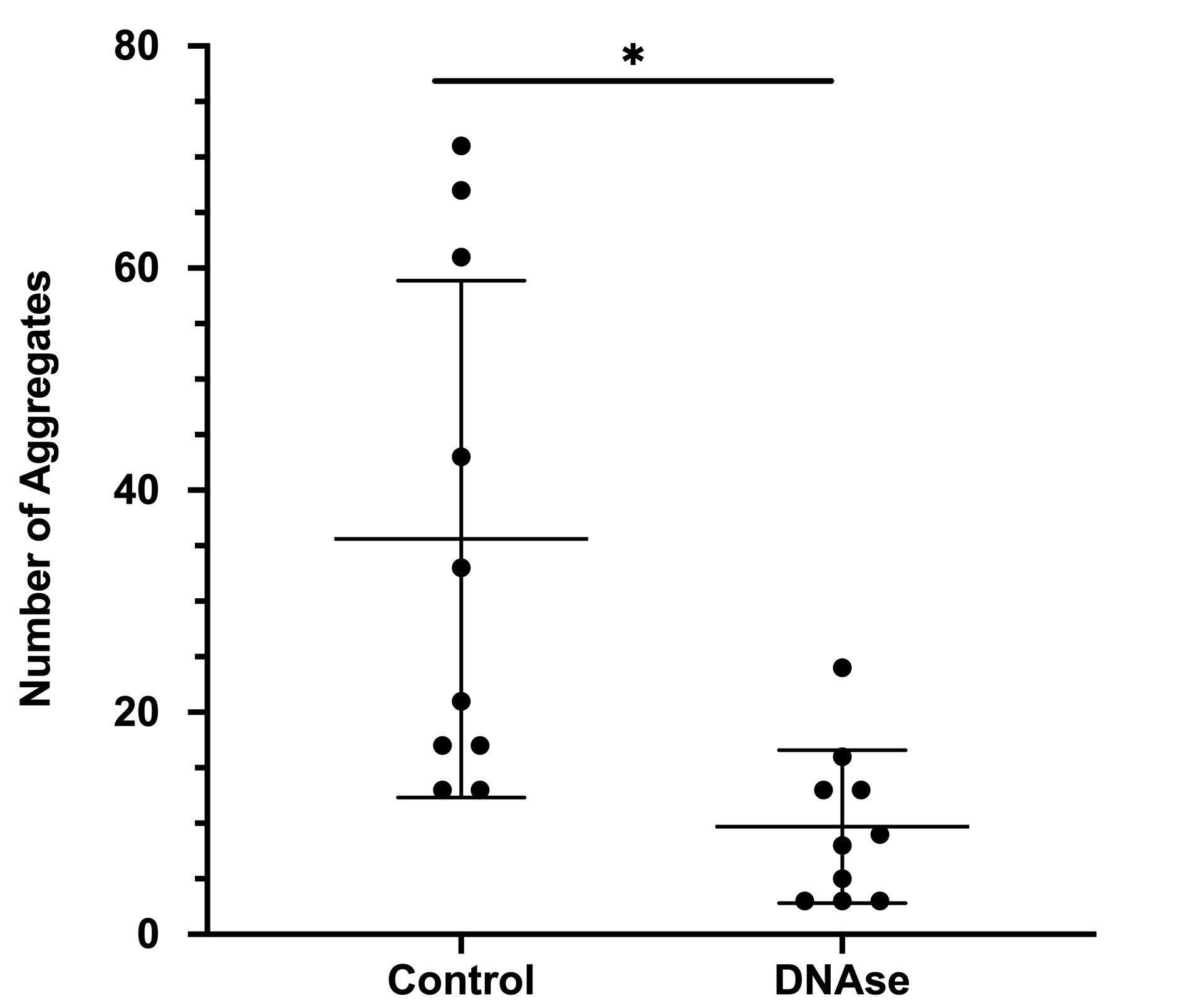


**Figure S3**. The number of tear PMNs aggregates counted before and after adding DNase. n = 10, *p* = 0.002.

**
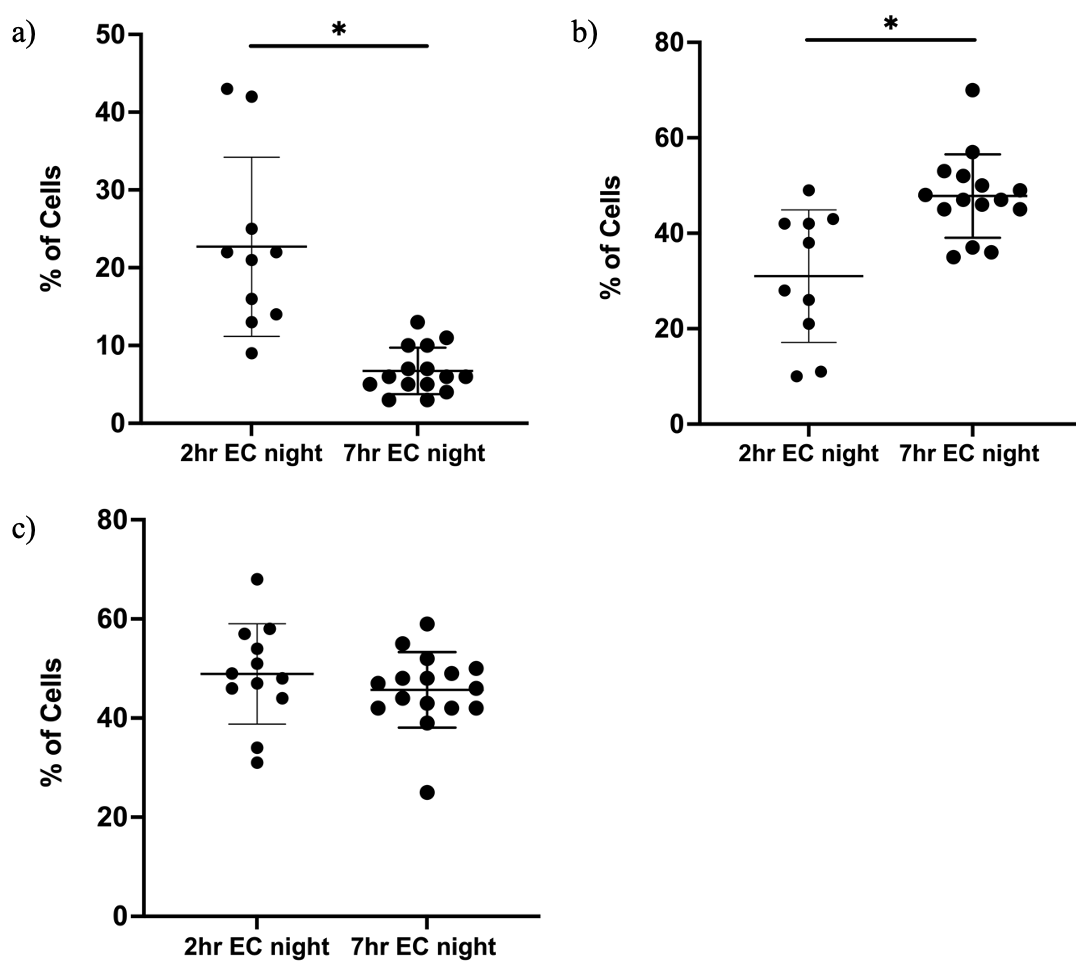
**

**Figure S4.** The percentages of a) hyposegmented, b) hypersegmented, and c) three lobed nuclei tear PMNs collected after 2hr and 7hr eye closures at night.


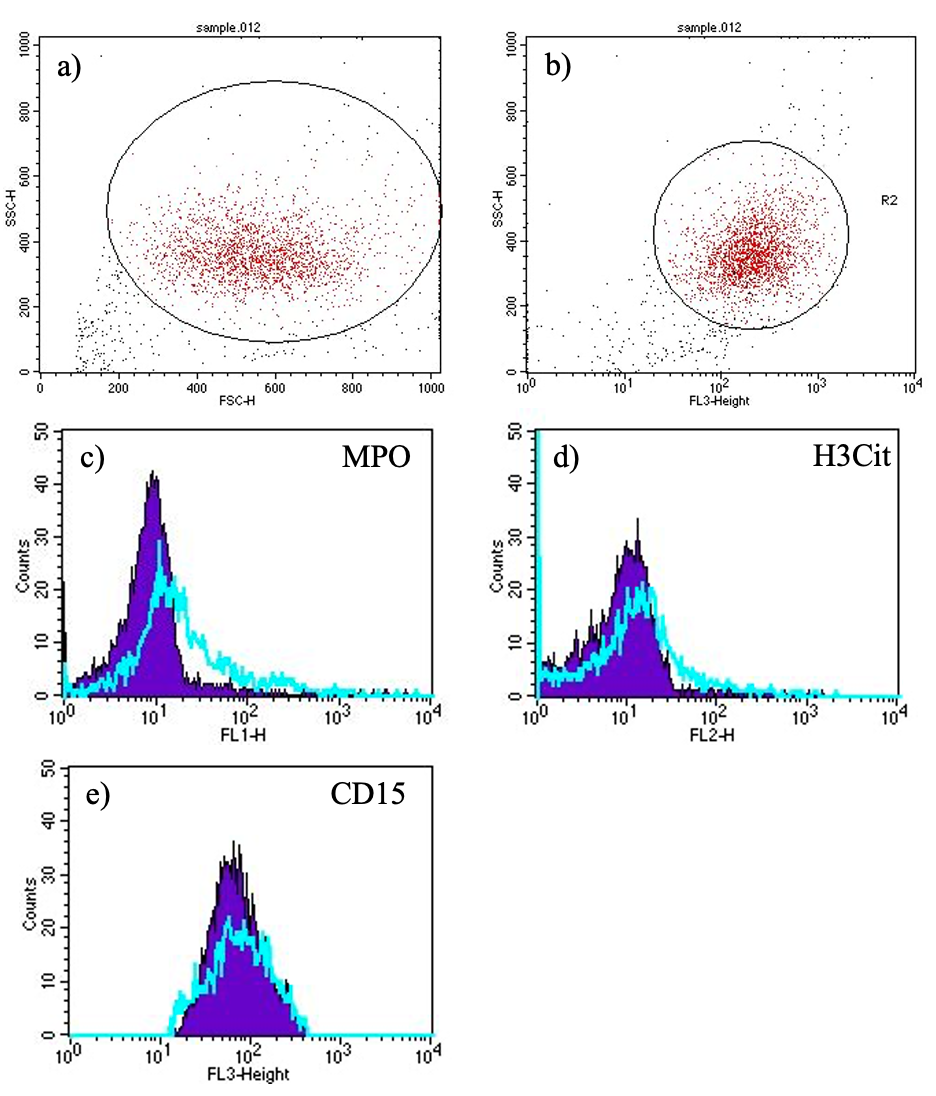


**Figure S5.** Measurement of NETs release by tear PMNs using flow cytometry analysis. Dot plots and histograms from 2hr (purple-filled) and 7hr (blue outlining) EC night tear PMNs. a) PMN population was identified based on their size (forward scatter, FSC) and cytoplasmic granularity (side scatter, SSC); b) side scatter versus CD15 (FL3) to identify the CD15 positive cells (i.e. neutrophils); c) the comparison of the MPO signals between 2hr and 7hr EC night tear PMNs on histogram plot; d) the comparison of the H3Cit (FL2) signals between 2hr and 7hr EC night tear PMNs on histogram plot; e) the comparison of the CD15 signals between 2hr and 7hr EC night tear PMNs on histogram plot.


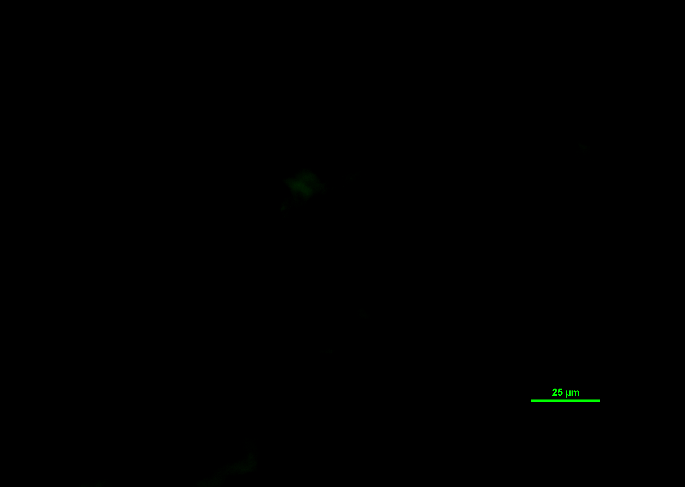

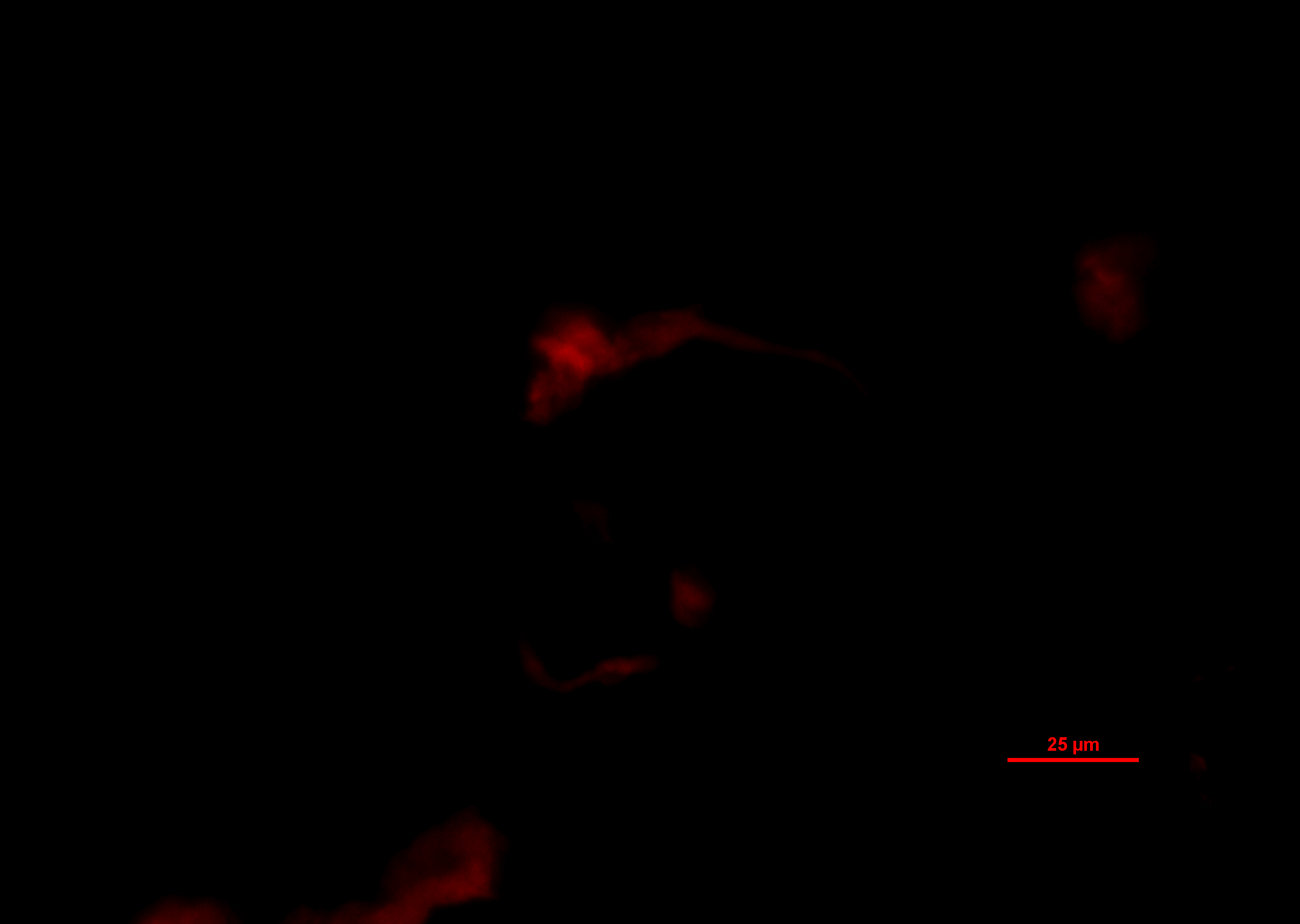

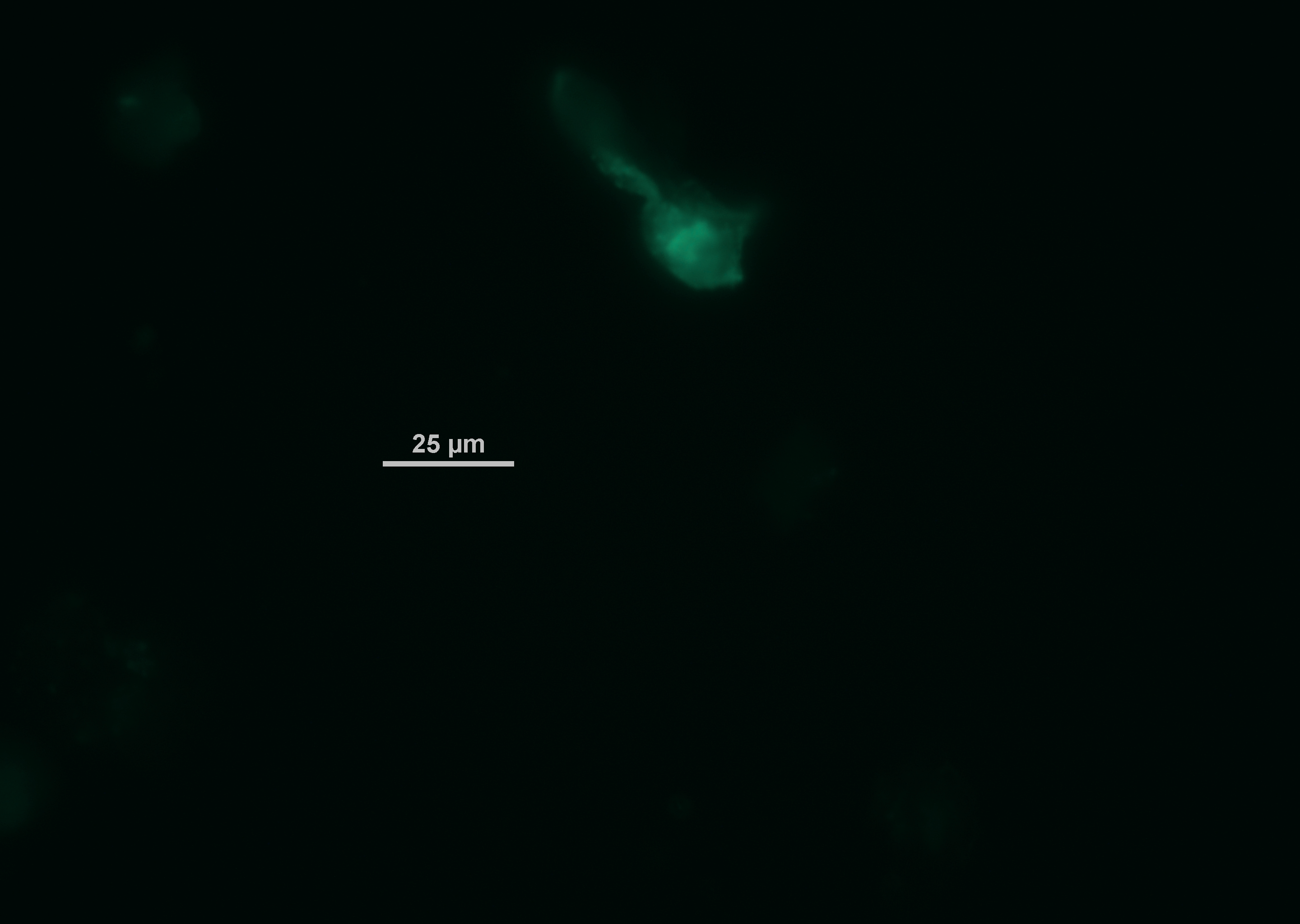

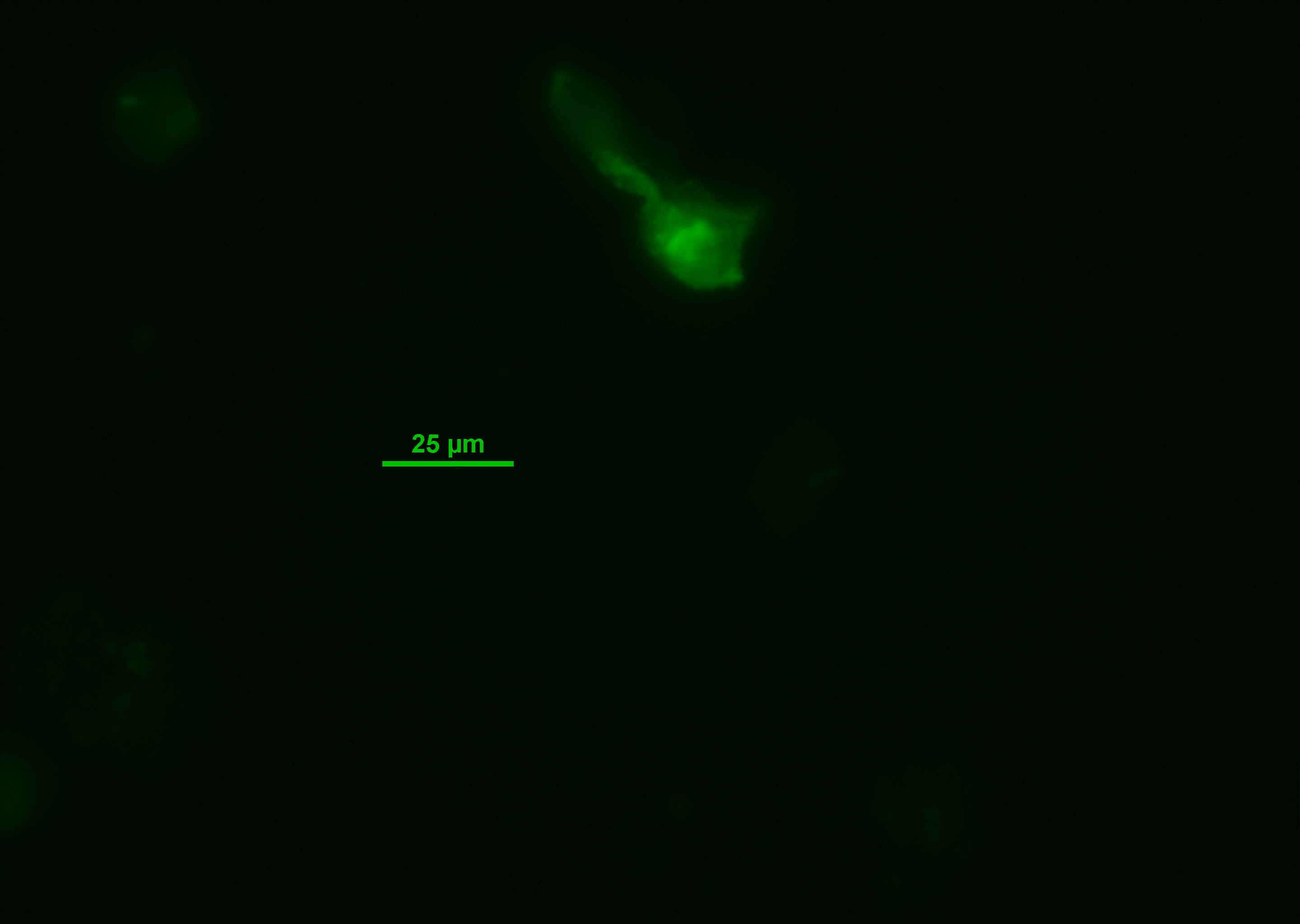

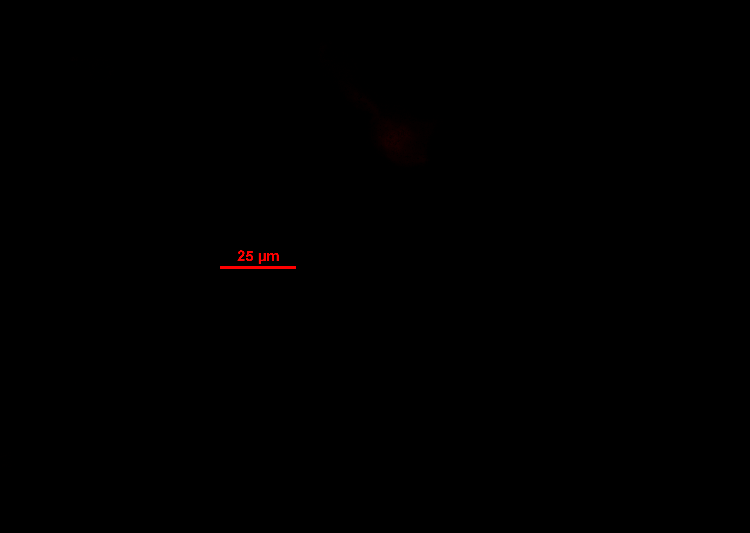

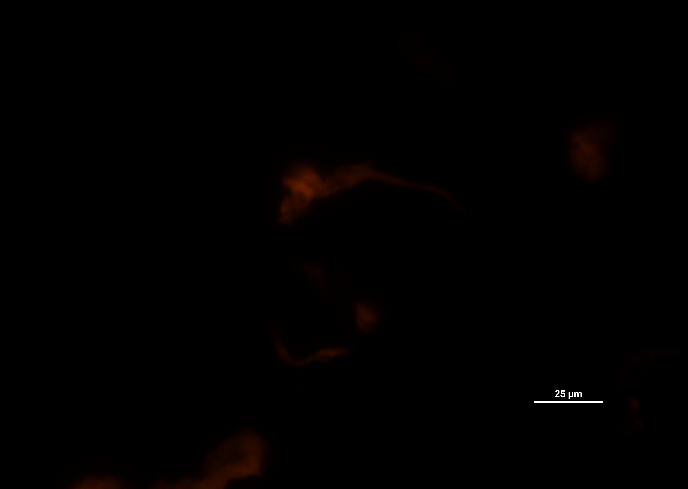


MPO

Green

H3Cit Red

Merged

a

d

b

e

c

f

**Figure S6**. Immunostaining controls of tear PMNs exhibiting NETs in tear samples collected after 7hr EC night. (a-c) a sample stained anti-citrullinated histones (H3Cit) antibody with its PE-conjugated secondary antibody, (d-f) a sample stained with FITC-conjugated MPO, a & d) green channel, b & e) red channel; c & f) merged fluorescence.

**Table S1.** The mean fluorescent values of CD62L, CD11b, and CD54 on 2hr and 7hr EC night tear PMNs.

|  | CD62L | CD11b | CD54 |
| --- | --- | --- | --- |
| 2hr EC night tear PMNs | 8.4 ± 2.1 | 132.6 ± 36.0 | 154.4 ± 34.6 |
| 7hr EC night tear PMNs | 8.1 ± 1.4 | 155.6 ± 44.5 | 176.4 ± 35.9 |

n = 5 for CD11b and CD54, and n = 10 for CD62L.

**Table S2.** The mean fluorescent values of lactoferrin expression on blood PMNs and 7hr EC and 2hr EC night tear PMNs.

|  | Unstimulated | fMLP-stimulated | Ratio of stimulated vs unstimulated |
| --- | --- | --- | --- |
| Blood PMNs | 40.8 ± 22.6 | 90.2 ± 66.2 | 2.2 ± 0.5 |
| 7hr EC night tear PMNs | 285.5 ± 107.5 | 323.7 ± 199.6 | 1.2 ± 0.3 |
| 2hr EC night tear PMNs | 120.0 ± 81.7 | 172.9 ± 105.0 | 1.4 ± 0.4 |

n = 5 for blood PMNs, n = 11 for 7hr EC night tear PMNs, and n = 8 for 2hr EC night tear PMNs.
